# High levels of biomarkers of collagen remodeling are associated with increased mortality in COPD – results from the ECLIPSE study

**DOI:** 10.1186/s12931-016-0440-6

**Published:** 2016-10-04

**Authors:** Jannie M. B. Sand, Diana J. Leeming, Inger Byrjalsen, Asger R. Bihlet, Peter Lange, Ruth Tal-Singer, Bruce E. Miller, Morten A. Karsdal, Jørgen Vestbo

**Affiliations:** 1 Nordic Bioscience, Herlev, Denmark; 2 Section of Social Medicine, Institute of Public Health, University of Copenhagen, Copenhagen, Denmark; 3 Section of Respiratory Medicine, Hvidovre Hospital, Hvidovre, Denmark; 4 Respiratory Therapy Area Unit, GSK Research and Development, King of Prussia, PA USA; 5 Centre for Respiratory Medicine and Allergy, Manchester Academic Science Centre, The University of Manchester and University Hospital South Manchester NHS Foundation Trust, Manchester, UK

**Keywords:** Extracellular matrix, Remodeling, Collagen, Elastin, Biomarker, COPD, Mortality, Prognostic

## Abstract

**Background:**

There is a need to identify individuals with COPD at risk for disease progression and mortality. Lung tissue remodeling is associated with the release of extracellular matrix (ECM) fragments into the peripheral circulation. We hypothesized that ECM remodeling was associated with mortality in COPD and measured neo-epitopes originating from ECM proteins associated with lung tissue remodeling.

**Methods:**

Biomarkers of ECM remodeling were assessed in a subpopulation (*n* = 1000) of the Evaluation of COPD Longitudinally to Identify Predictive Surrogate End-points (ECLIPSE) cohort. Validated immunoassays measuring serological neo-epitopes produced by proteolytic cleavage associated with degradation of collagen type I, III, IV, and VI, elastin, and biglycan, and formation of collagen type VI as well as fibrinogen and C-reactive protein were used. Multivariate models were used to assess the prognostic value of these biomarkers.

**Results:**

Thirty subjects (3.0 %) died during follow-up. Non-survivors were older, had reduced exercise capacity, increased dyspnea score, and included fewer current smokers. All collagen biomarkers were significantly elevated in non-survivors compared to survivors. Mortality risk was significantly increased for subjects with collagen remodeling biomarkers in the upper quartile, especially for the degradation fragment of collagen type IV C6M (hazard ratio 6.6 [95 % confidence interval 2.9-15.2], *P* < 0.0001) after adjusting for relevant confounders.

**Conclusions:**

Serological biomarkers of collagen remodeling were strongly associated with mortality in subjects with COPD indicating that assessment of tissue turnover in the parenchyma and small airways may be useful in the prognosis of COPD.

**Trial registration:**

NCT00292552, GSK Study No. SCO104960.

**Electronic supplementary material:**

The online version of this article (doi:10.1186/s12931-016-0440-6) contains supplementary material, which is available to authorized users.

## Background

Chronic obstructive pulmonary disease (COPD) is a heterogeneous disease characterized by progressive airflow limitation and a chronic inflammatory response of the lungs. It is a major cause of morbidity and mortality and is estimated to be the third leading cause of death worldwide [1]. Poor lung function, lung function decline [2], low body mass index (BMI) [2], a high level of dyspnea [3], frequent exacerbations [4], and low levels of physical activity [5] have all been identified as predictors of mortality in individuals with COPD. Using multidimensional risk scores [6] or adding markers of systemic inflammation [7] adds to the predictive value, exceeding the prognostic value of lung function alone. The recent statement from the American Thoracic Society and the European Respiratory Society on research questions in COPD encourages the identification of surrogate markers of patient-centered outcomes such as quality of life and mortality [8]. Being able to further improve prediction of mortality would aid in the selection of patients for specific interventions and clinical trials and would ultimately improve personalized healthcare.

**Fig. 1:**
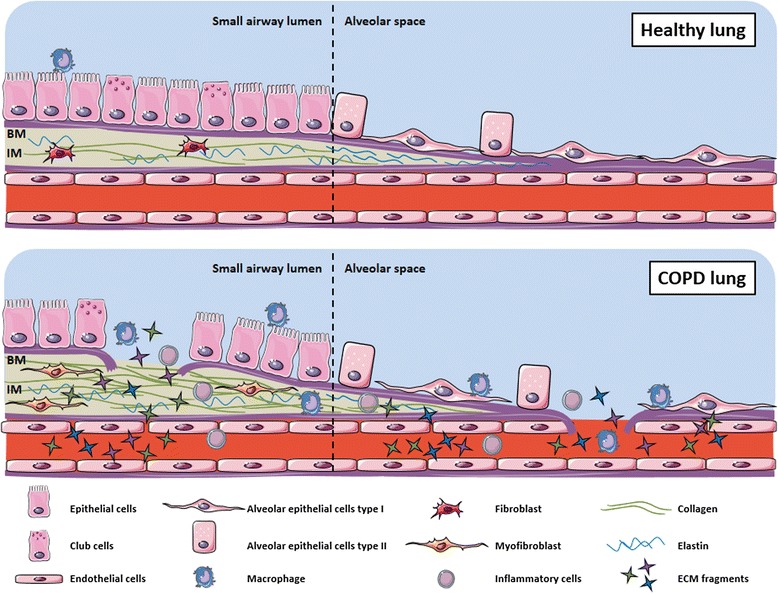
Extracellular matrix composition in healthy and COPD lungs. In healthy lungs, the epithelial cells create a tight barrier thereby blocking entry of foreign particles from the inhaled air to the tissue. This is further enforced by the underlying basement membrane (BM) which mainly consists of collagen type IV. The interstitial matrix (IM) is below the BM and consists mainly of fibrillar collagens and elastin. In COPD, the continuous epithelial layer is disrupted and the underlying BM is exposed. The inflammatory response to repetitive tissue injury results in up-regulation of proteases and disruption of the BM, allowing for injury to the underlying IM. In response to this, fibroblasts are activated and converted to pro-fibrotic myofibroblasts that secrete collagens which accumulate in the IM of the airway wall. Both collagens and elastin undergoes proteolytic degradation in the airway and alveolar walls. The processes of synthesis and degradation release extracellular matrix (ECM) fragments which may enter the bloodstream from where they can be assessed as biomarkers of ECM remodeling

The chronic inflammatory response in the airways of individuals with COPD is associated with increased numbers of inflammatory cells and fibroblasts and the up-regulation of proteases such as matrix metalloproteinases (MMPs) resulting in fibrosis of small airways and destruction of the parenchyma [9]. The altered microenvironment affects the composition of the extracellular matrix (ECM), mainly comprised of collagens and elastin [10]. The two main extracellular compartments of the lungs are the basement membrane and the interstitial matrix (Fig. 1). The main constituent of the basement membrane is collagen type IV, whereas the interstitial matrix is rich in collagens type I and III and elastin. The chronic inflammatory process in the lungs of individuals with COPD induces the remodeling of the ECM, which includes both degradation of old proteins and synthesis of new ones and occurs in both small airways and alveolar walls. This results in the release of protein fragments or neo-epitopes into the systemic circulation where they can be assessed as biomarkers of lung ECM remodeling (Fig. 1). C1M, C3M, C3A, and C6M are biomarkers of collagen type I, III and VI degradation, respectively, whereas Pro-C6 assesses the C5 domain of collagen type VI believed to be released during protein maturation [11–14]. C4M is a biomarker of collagen type IV degradation mediated by MMPs and thus assesses basement membrane destruction [15]. EL-NE and ELM7 are biomarkers of neutrophil elastase- and MMP-7-mediated degradation of elastin, a protein crucial for the elastic recoil of the lungs [16, 17]. The proteoglycan biglycan is a key player in the assembly of collagen fibrils and its degradation by MMPs can be assessed by the biomarker BGM [18]. The biomarker CRPM assesses the local inflammation by measuring MMP-mediated degradation of C-reactive protein (CRP) [19].

As excessive ECM remodeling resulting from repetitive injury is associated with COPD [20], we hypothesized that measuring serological biomarkers of ECM protein remodeling would be helpful in predicting all-cause mortality in individuals with COPD. We investigated this by assessing biomarkers of ECM degradation and formation in a subpopulation of the Evaluation of COPD Longitudinally to Identify Predictive Surrogate End-points (ECLIPSE) cohort. We included analyses of the previously studied CRP and fibrinogen [7] for comparison of prognostic performance.

## Methods

### Study design

The study design of ECLIPSE (clinicaltrials.gov identifier NCT00292552; GSK study code SCO104960) has been described in detail previously [21, 22]. Briefly, ECLIPSE is an observational, longitudinal study where participants were evaluated at baseline and at months three and six, and subsequently every six months for three years. For these analyses, we used clinical and biomarker data obtained at baseline, month six and year one. Death was determined up to day 1060 of the study. Cause of death was not available for the present analyses. ECLIPSE complies with the Declaration of Helsinki and Good Clinical Practice Guidelines, and has been approved by the ethics committees of the participating centers. All participants provided written informed consent before the performance of any study-related assessments.

### Population

The full ECLIPSE study included 2164 subjects with COPD based on a post-bronchodilator forced expiratory volume in one second (FEV_1_) < 80 % of the reference value, FEV_1_/forced vital capacity (FVC) ≤ 0.7, and a smoking history of greater than or equal to 10 pack-years [21]. The current analyses were performed on a sub-population of 1000 COPD subjects of the ECLIPSE cohort composed of the 500 subjects that progressed the most and the 500 subjects that progressed the least in terms of FEV_1_ decline over the three year study period. Participants were recruited from the outpatient clinics of the participating centers. Significant emphysema was defined as low attenuation area at −950 Hounsfield Units (%LAA) ≥ 10 % on chest computed tomography (CT) scans. Non-emphysematous COPD was defined as %LAA < 5 %.

### Measurements of serological biomarkers

Whole blood was collected by venipuncture into vacutainer tubes from participants in the fasting state. Serum was prepared by allowing the blood to clot for 30 min at room temperature followed by centrifugation at 1500 g for 10–15 min. Plasma was obtained by centrifugation of vacutainer tubes at 2000 g for 10–15 min. Serum and plasma were stored at −80 °C until analyzed and did not undergo any freeze/thaw cycles prior to measurements of the current biomarkers. C1M, BGM, EL-NE, and Pro-C6 were measured in month six serum samples, while C3A, C3M, C4M, C6M, ELM7, and CRPM were measured in year one plasma (heparin anticoagulant) samples. Details of these biomarkers are presented in Table 1. Prior to this study, fibrinogen and CRP (high-sensitivity method) were measured in year one plasma (ethylenediaminetetraacetic acid anticoagulant) samples as described previously [7]. All biomarkers were measured by validated competitive ELISAs utilizing monoclonal antibodies targeting specific neo-epitopes (Nordic Bioscience, Herlev, Denmark) [11–19] and measurements were performed in a blinded manner according to the manufacturer’s instructions. Analytes detected by these assays are stable in serum/plasma samples that have undergone at least four freeze/thaw cycles [11–19].

**Table 1:** Biomarker specifications

Biomarker	Specifications	Measure	References
BGM	Biglycan degraded by MMPs	IM remodeling	[18]
C1M	Collagen type I degraded by MMPs	IM remodeling	[11]
C3A	Collagen type III degraded by ADAMTS	IM remodeling	Unpublished^a^
C3M	Collagen type III degraded by MMP	IM remodeling	[12]
C4M	Collagen type IV degraded by MMPs	BM remodeling	[15]
C6M	Collagen type VI degraded by MMPs	IM remodeling	[13]
CRPM	CRP degraded by MMPs	Local chronic inflammation	[19]
ELM7	Elastin degraded by MMP-7	IM remodeling	[17]
EL-NE	Elastin degraded by neutrophil elastase	IM remodeling	[16]
Pro-C6	Collagen type VI C5 domain (released)	IM remodeling	[14]

*MMP* matrix metalloproteinase, *ADAMTS* a disintegrin and metalloproteinase with thrombospondin motifs, *IM* interstitial matrix, *BM* basement membrane, *CRP* C-reactive protein

^a^The C3A assay was validated following a standard protocol as described in [11]

### Statistical analysis

Survivor and non-survivor characteristics were compared using student’s *t*-test, chi-squared test, or Mann–Whitney *U* test as appropriate. Serological biomarker data were logarithmically transformed to obtain normality and are shown as geometric mean with standard error of mean (SEM). Biomarker levels were compared between groups with student’s *t*-test. Cox proportional hazards analyses were used to assess the prognostic value of each biomarker for all-cause mortality for a one standard deviation increase in biomarker and comparing the upper and lower biomarker quartiles. The risk of death was assessed using crude analyses and analyses adjusted for age, BODE index (added as categorical variable), and number of exacerbations in the year prior to baseline measurements. The discriminative power of each serological biomarker, the clinical model (age, BODE, and previous exacerbations), and the clinical model with additions of one or more serological biomarker were assessed by receiver operating curve (ROC) analyses. Area under the ROC curve (AUC) for the clinical model was compared with that of the clinical model with added serological biomarkers by the method of Delong et al. 1988 [23]. All tests performed (SAS version 9.3, SAS Institute Inc., Cary, NC, USA; MedCalc version 14.8.1, MedCalc Software bvba, Ostend, Belgium) were two-sided at the 0.05 level of significance, and all *P* values are nominal as no adjustments were made for multiple comparisons.

## Results

### Basic demographics

Of the 1000 subjects included, 985 (99 %) were Caucasians, 363 (36 %) were female, and mean age was 63 (SD 7) years. BGM, EL-NE, and ProC6 were significantly related to gender with mean levels in male vs. female of 46.09 (95 % CI 44.52–47.70) ng/mL vs. 42.04 (95 % CI 40.29–43.88) ng/mL (*p* = 0.0013), 8.23 (95 % CI 7.77–8.71) ng/mL vs. 7.15 (95 % CI 6.67–7.67) ng/mL (*p* = 0.0029), and 6.51 (95 % CI 6.35–6.67) ng/mL vs. 6.25 (95 % CI 6.06–6.45) ng/mL (*p* = 0.048), respectively.

Thirty subjects (3.0 %) died during the follow-up period of a minimum of two years from time of blood sampling. Table 2 shows the baseline characteristics of survivors and non-survivors. Non-survivors were significantly older, reported more dyspnoea, lower fatigue scores, had reduced 6MWD, and fewer subjects were current smokers. Severity of airflow limitation, comorbidity profile, presence of emphysema on high-resolution computed tomography scan, BODE index, number of exacerbations, and medications were not significantly different between survivors and non-survivors.

**Table 2:** Subject characteristics at baseline

	Survivors	Non-survivors	*P* value
	(*n* = 970)	(*n* = 30)	
Demographics			
Age (yrs)*	63 ± 7	68 ± 6	<0.0001
Female gender^a^	352 (36)	11 (37)	0.97
BMI (kg/m^2^)*	26.8 ± 5.8	27.3 ± 7.8	0.78
Current smoker (%)^a^	366 (38)	3 (10)	0.004
Pack years (yrs)*	47 ± 25	56 ± 42	0.27
Clinical variables			
FEV_1_ (L)*	1.42 ± 0.51	1.27 ± 0.43	0.11
FEV_1_ (% predicted)*	51 ± 15	48 ± 13	0.44
GOLD Stage^b^			
II	480 (49)	14 (47)	0.93
III	392 (40)	14 (47)	
IV	98 (10)	2 (7)	
Number of previous exacerbations^b^			
0	531 (55)	20 (67)	0.29
1	239 (25)	5 (17)	
2	116 (12)	2 (7)	
> 2	84 (9)	3 (10)	
6MWD (meters)*	386 ± 119	335 ± 105	0.02
mMRC dyspnoea score; median (Q1; Q3) ^b^	1 (1;2)	2 (1;3)	0.02
%LAA (%)*	16.3 ± 11.3	17.4 ± 10.5	0.61
BODE index; median (Q1; Q3) ^b^	3 (1;4)	3.5 (2;5)	0.06
SGRQ total score*	46 ± 18	50 ± 20	0.29
FACIT fatigue score*	36 ± 10	31 ± 12	0.01
Comorbidities^a^			
Cardiovascular history	304 (31)	12 (40)	0.42
Hypertension	381 (39)	14 (47)	0.53
Asthma history	225 (23)	8 (27)	0.82
Diabetes type II	82 (8)	3 (10)	0.97
Osteoarthritis	125 (13)	3 (10)	0.85
Osteoporosis	121 (12)	6 (20)	0.35
Rheumatoid arthritis	29 (3)	0 (0)	0.68
Inflammatory bowel disorder	48 (5)	1 (3)	0.98
Interventions^a^			
Inhaled corticosteroids	140 (14)	3 (10)	0.68
Systemic corticosteroids	8 (0.8)	0 (0)	0.59
Statins	237 (24)	6 (20)	0.73

Data are shown as mean ± SD or n (%) at baseline unless otherwise stated

*BMI* body mass index, *FEV*_*1*_ postbronchodilator forced expiratory volume in one second, *GOLD* global initiative for chronic obstructive lung disease, *6MWD* 6-min walk distance, *mMRC* modified Medical Research Council dyspnoea scale, *%LAA* low attenuation area at −950 Hounsfield Units, *BODE* BMI, airflow obstruction, dyspnea and exercise capacity index, *SGRQ* St George’s respiratory questionnaire, *FACIT* functional assessment of chronic illness therapy

Statistical significance was determined using student’s *t*-test (*), chi-squared test (^a^), or Mann–Whitney *U* test (^b^)

### ECM remodeling related to emphysema

At year one, 624 subjects presented with significant emphysema, defined as a %LAA ≥10 %, while 147 subjects showed no signs of emphysema on chest CT scan, defined as %LAA <5 %. Mean %LAA for subjects with and without emphysema was 22.8 % (SD 9.9) and 3.1 % (SD 1.3), respectively. CRPM was the only biomarker that differed significantly between emphysematous and non-emphysematous COPD, with levels of 9.37 (95 % CI 9.09–9.65) ng/mL and 10.11 (95 % CI 9.43–10.84) ng/mL, respectively (*p* = 0.032).

Of the subjects with significant emphysema, 550 had data available at year three and had a mean change in %LAA from year one of +5.5 % (SD 22.3). Subjects with increased %LAA (emphysema progressors), had significantly lower levels of C1M, EL-NE, and ProC6 than those with a negative or no change in %LAA (emphysema non-progressors) (C1M: 68.85 ng/mL (95 % CI 64.74–73.21) vs. 77.67 ng/mL (95 % CI 71.31–84.61), *p* = 0.022; EL-NE: 7.34 ng/mL (95 % CI 6.81–7.91) vs. 8.41 ng/mL (95 % CI 7.64–9.25), *p* = 0.028; ProC6: 6.16 ng/mL (95 % CI 5.98–6.34) vs. 6.52 ng/mL (95 % CI 6.25–6.81), *p* = 0.032).

### ECM remodeling related to mortality

**Fig. 2:**
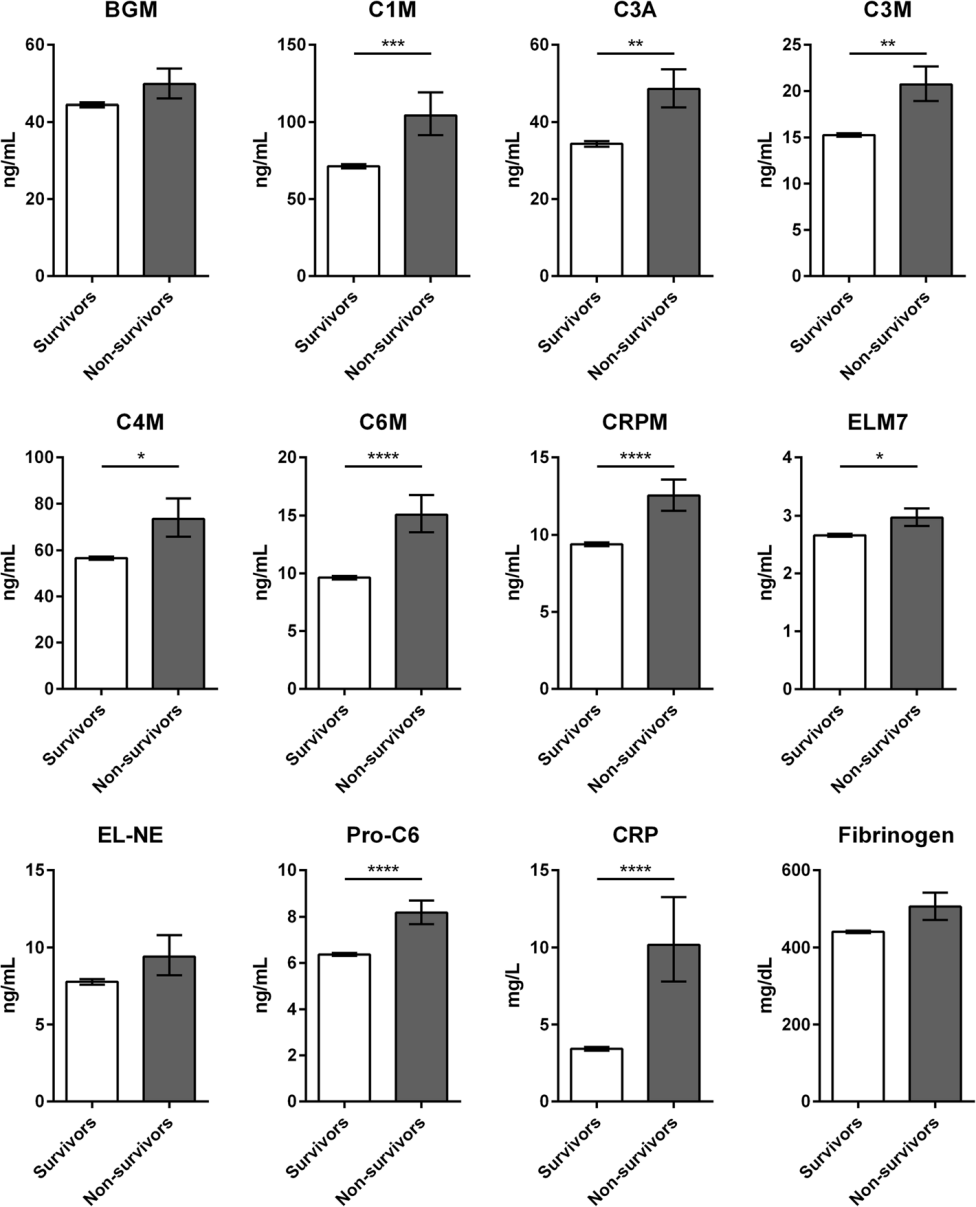
Biomarker levels in survivors versus non-survivors. Biomarker levels were assessed at month six (BGM, C1M, EL-NE, Pro-C6) or year one (C3A, C3M, C4M, C6M, CRPM, ELM7, CRP, fibrinogen) in survivors (*n* = 970) and non-survivors (*n* = 30). All biomarkers, with the exception of BGM, EL-NE, and fibrinogen, were significantly elevated in non-survivors compared to survivors. Data are shown as geometric mean ± SEM. Statistical significance was determined using student’s *t*-test: *p < 0.05; **p < 0.01; ***p < 0.001; ****p < 0.0001

Eight of the ten serological biomarkers of ECM remodeling (C1M, C3A, C3M, C4M, C6M, ProC6, ELM7, and CRPM, but not EL-NE and BGM) were significantly elevated in subjects who died compared to survivors (Fig. 2). Plasma CRP, but not fibrinogen, was also significantly elevated in non-survivors (Fig. 2).

**Fig. 3:**
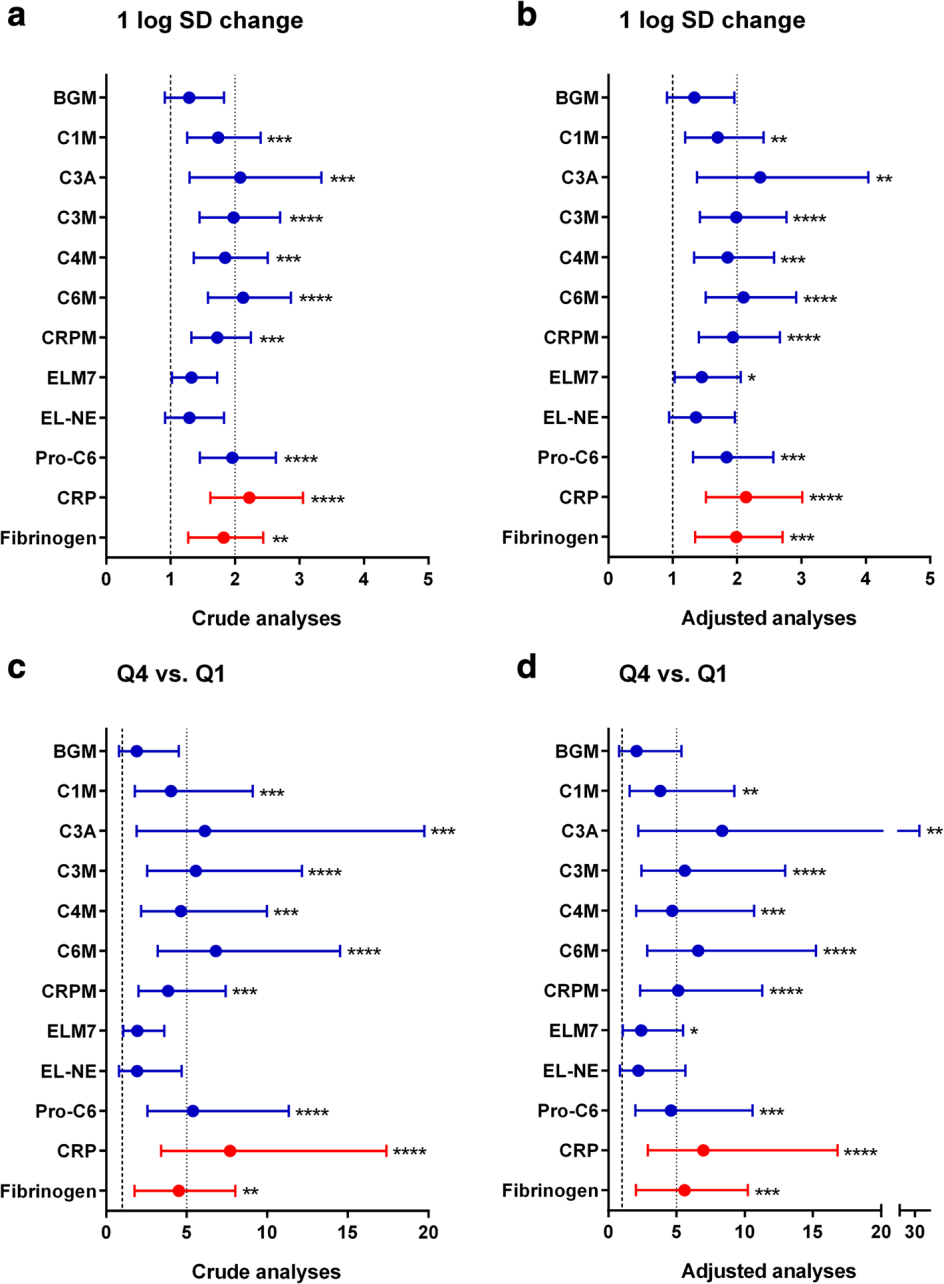
Cox proportional hazards ratios for death. Hazard ratios (HR) for death are shown per one standard deviation increase in biomarker (**a** + **b**) and for subjects in the upper vs. lower quartile (**c** + **d**). Cox proportional HR with 95 % confidence intervals are shown for crude analyses (**a** + **c**) and analyses adjusted for age, BODE, and previous exacerbations (**b** + **d**). All biomarkers, with the exception of BGM, ELM7, and EL-NE, were significantly related to mortality outcome in both crude and adjusted analyses. Statistical significant hazard ratios are indicated as *p < 0.05; **p < 0.01; ***p < 0.001; ****p < 0.0001

Figure 3 shows the crude and adjusted (age, BODE, and previous exacerbations) Cox proportional hazards ratios for death within the follow-up period. The ECM remodeling biomarker C6M was most prognostic for all-cause mortality with crude and confounder-adjusted hazard ratios of 6.8 (95 % CI 3.2–14.5, *P* < 0.0001) and 6.6 (95 % CI 2.9–15.2, *P* < 0.0001), respectively, for subjects belonging to the upper vs. lower biomarker quartile. CRP performed equally well with hazard ratios of 7.7 (95 % CI 3.4–17.4, *P* < 0.0001) and 7.0 (95 % CI 2.9–16.8, *P* < 0.0001), respectively. C1M, C3A, C3M, C4M, CRPM, Pro-C6, and fibrinogen were also prognostic for mortality in both crude and adjusted analyses. Further adjustment for variables that were significantly different between survivors and non-survivors (Table 2) resulted in similar hazard ratios (data not shown).

**Fig. 4:**
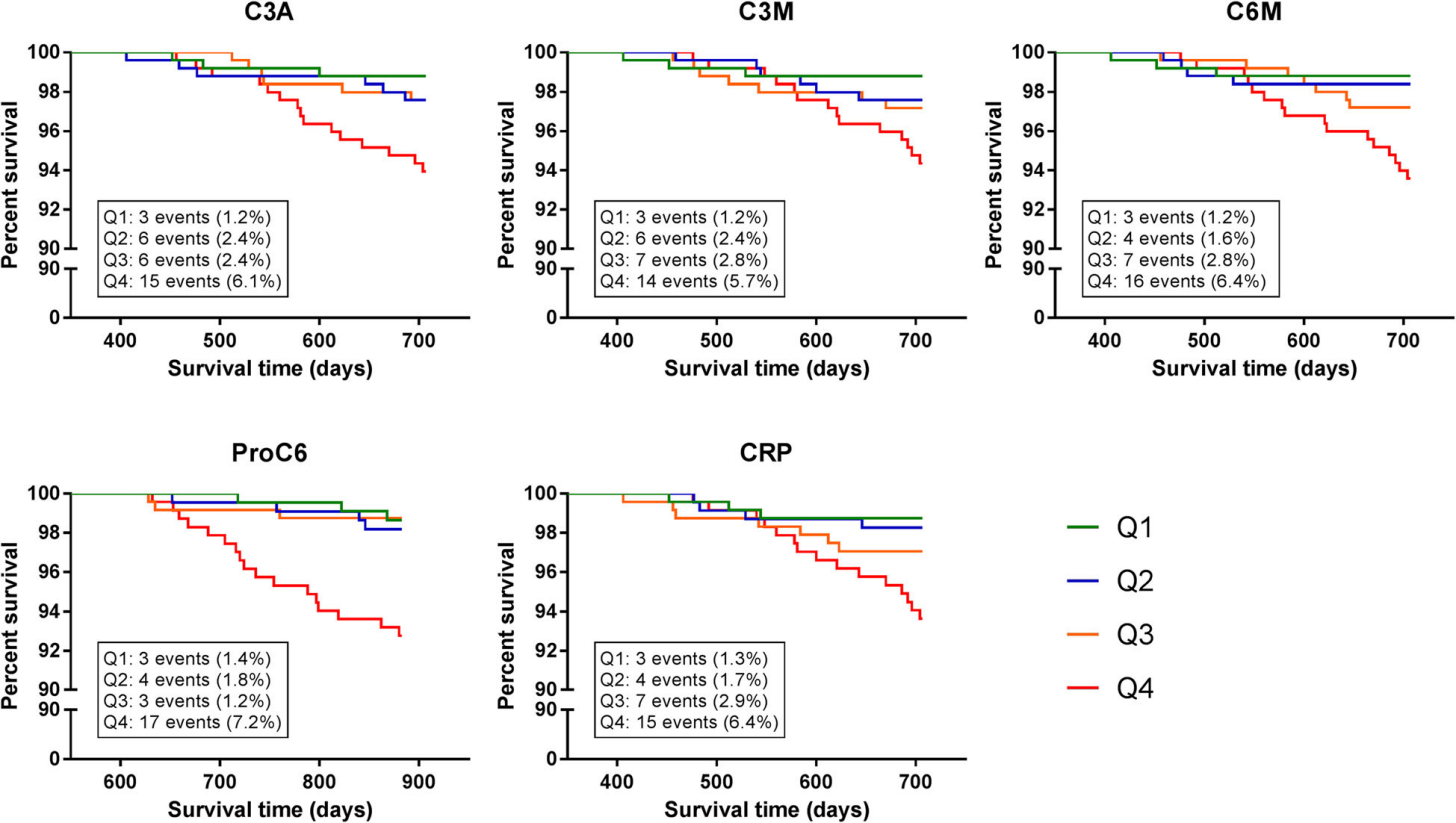
Kaplan-Meier survival curves for biomarker quartiles. Kaplan-Meier survival curves are shown for the biomarkers with crude hazard ratios for quartile 4 vs. 1 above 5. The relationship of biomarker quartiles with survival time from blood sampling are shown for plasma C3A (year one), plasma C3M (year one), plasma C6M (year one), serum ProC6 (month six), and plasma CRP (year one). Subjects in the upper biomarker quartiles (Q4) showed a higher number of deaths within the study period than the lower quartiles

Kaplan-Meier survival curves for the biomarkers with crude hazard ratios above 5.0 (C3A, C3M, C6M, Pro-C6, CRP) are shown in Fig. 4. For all five biomarkers, being in the upper quartile was associated with an increased number of deaths. In the upper quartile of C6M, 16 out of 249 (6.4 %) subjects died, while only 3 of 249 (1.3 %) died in the lower quartile. For Pro-C6, the similar numbers were 17 out of 235 (7.2 %) and 3 out of 223 (1.4 %), respectively. By selecting subjects belonging to the upper quartile of both C6M and Pro-C6, we identified 10 subjects (11.1 %) that died during the follow-up period out of the 90 subjects that were in the upper quartile for C6M and Pro-C6.

The discriminatory power for predicting death of the serological biomarkers alone and a clinical model with added serological biomarkers was assessed by ROC analyses (Table 3). The AUC for the clinical model (including age, BODE, and previous exacerbations) was 0.750 (95 % CI 0.721–0.778) and the addition of one or more biomarkers to this model increased the AUC to 0.871 (95 % CI 0.846–0.893, *P* = 0.0004) for the addition of C6M, Pro-C6, and CRP.

**Table 3:** Discriminatory power for the prognosis of death

	AUC (95 % CI)	*P* value
Serological biomarkers		
C6M	0.724 (0.695–0.752)	-
Pro-C6	0.722 (0.692–0.751)	-
CRP	0.719 (0.689–0.747)	-
C6M + Pro-C6	0.796 (0.769–0.822)	-
C6M + CRP	0.727 (0.697–0.755)	-
Pro-C6 + CRP	0.775 (0.746–0.802)	-
C6M + Pro-C6 + CRP	0.791 (0.762–0.818)	-
Clinical models		
Age + BODE + Previous exacerbations	0.750 (0.721–0.778)	
+ C3M	0.803 (0.776–0.829)	0.084
+ CRPM	0.807 (0.780–0.832)	0.044
+ C6M	0.813 (0.786–0.837)	0.038
+ Pro-C6	0.826 (0.799–0.851)	0.071
+ CRP	0.811 (0.783–0.836)	0.046
+ C6M + Pro-C6	0.867 (0.843–0.889)	0.001
+ C6M + CRP	0.817 (0.790–0.842)	0.023
+ C6M + CRPM	0.818 (0.791–0.842)	0.036
+ Pro-C6 + CRP	0.863 (0.837–0.886)	0.001
+ Pro-C6 + CRPM	0.864 (0.840–0.886)	0.003
+ C6M + Pro-C6 + CRP	0.871 (0.846–0.893)	0.0004
+ C6M + Pro-C6 + CRPM	0.871 (0.847–0.893)	0.001

Shown are the single biomarkers with an area under the ROC curve (AUC) >0.700 and models with AUC >0.800. P values compare the AUC of the clinical model alone to the clinical model with added biomarker(s)

## Discussion

We have shown for the first time that elevated levels of serological biomarkers of ECM remodeling were associated with increased mortality in subjects with COPD. Several novel biomarkers of collagen degradation were significant predictors of mortality within two years of follow-up in a subpopulation of the ECLIPSE cohort. Results for biomarkers of ECM remodeling (collagen type I (C1M), III (C3A, C3M), IV (C4M), and VI (C6M) degradation and collagen type VI formation (Pro-C6)), strongly suggest that remodeling of the interstitial collagens is of high importance in COPD.

In individuals with COPD, the remodeling of the lung ECM is altered as a consequence of the inflammatory process and the delicate balance between protein formation and degradation is shifted, affecting both the structure and function of the tissue [24]. In both alveolar and small airway walls of individuals with COPD, fibrillar collagen content, as well as collagen type VI, has been found to be increased while elastin content was decreased, compared to controls [25, 26]. A preliminary study assessing biomarkers of ECM turnover in ten subjects with mild COPD found elevated levels of collagen neo-epitopes as compared to healthy controls, indicating an excessive ECM remodeling already in mild COPD [27]. Elevated levels of biomarkers of ECM degradation during exacerbations of COPD as compared to the stable state furthermore indicate a relation of the rate of ECM turnover to disease activity [28, 29]. However, these smaller studies lack long-term follow-up.

Most likely, initial injury to the airways and lung parenchyma disrupts the continuous epithelial cell layer and exposes the underlying basement membrane, which mainly consists of collagen type IV. The subsequent destruction of the basement membrane by MMPs, up-regulated in response to injury, release fragments of collagen type IV (C4M) to the circulation. Deeper and chronic tissue damage expose the underlying interstitial matrix, consisting mainly of collagen type I and III, to tissue remodeling, resulting in the destruction of the interstitial matrix and the release of fragments of collagen type I (C1M), III (C3A and C3M), and VI (C6M). The repetitive tissue injury is believed to increase ECM remodeling rate and shift the balance of synthesis and degradation, favoring collagen deposition in small airway walls (fibrosis) and destruction of collagens and elastin in alveolar walls (emphysema) [10] (Fig. 1). By measuring MMP-mediated protein degradation, the local inflammatory process is included in the assessments. This is true for the collagen, elastin and CRP biomarkers assessed here. The alterations in lung ECM in relation to COPD lead to the release of specific protein neo-epitopes to the systemic circulation, thus allowing for the assessment of tissue remodeling by use of serological biomarkers [30] (Fig. 1).

In the present study, we found that biomarkers of the structural changes occurring in both the basement membrane and the interstitial matrix of the lungs were related to mortality in individuals with COPD. In the largest biomarker study in idiopathic pulmonary fibrosis to date, remodeling of the interstitial matrix and proteolytic degradation of CRP was also shown to be related to disease progression and mortality [31]. Consequently, the remodeling of the interstitial matrix is of paramount importance for both lung diseases encompassing fibrotic lesions. Our data indicate that collagen remodeling resulting in destruction of alveolar walls and both destruction and thickening of the small airway walls may be assessed by serological biomarkers, the levels of which are associated with mortality in COPD. Interestingly, we found no association of mortality with markers of elastin degradation, although an association to all-cause mortality has previously been reported for desmosine, another marker of elastin degradation [32]. This difference may be due to the neo-epitope specificity of the present biomarkers, which require specific protease activity in a specific location, or a difference in clearing from the blood for different elastin fragments. Interestingly, especially the remodeling of collagen type VI was shown to be associated with mortality in COPD in the current study. Collagen type VI is a unique beaded filament type of collagen found in the interstitial matrix near the basement membrane. It forms a microfibrillar network believed to anchor basement membranes, collagen fibers and cells to the connective tissue [33–35]. Furthermore, it may act as an early sensor of the injury/repair response as well as a regulator of fibrogenesis by modulating cell-cell interactions, stimulating proliferation of mesenchymal cells, and preventing cell apoptosis [36, 37]. The expression of collagen type VI has been found increased in fibrotic lungs [38] and its C5 domain, also known as endotrophin, has been shown to trigger adipose tissue fibrosis [39]. These functions point to an important role for collagen type VI in the development of fibrosis and may indicate a crucial function of this structural and functionally important collagen type in COPD as well.

Previous studies of serological biomarkers for the prediction of outcomes in individuals with COPD have mainly focused on the systemic inflammatory response by evaluating biomarkers such as interleukin-6, CRP and fibrinogen. However, plasma CRP levels shows a wide variability in individuals with stable COPD [40], making it less suitable as a prognostic biomarker for individual patients. Several of the biomarkers assessed here have previously been found to have low variability over a six months period in individuals with idiopathic pulmonary fibrosis [31]. Neo-epitopes released in relation to ECM remodeling are the “end products” of the local tissue inflammation and repair processes in the lungs [41], which might explain the relatively stable measurements of these biomarkers over time. However, ECM neo-epitopes are not exclusively released from lung tissue. A continuous low rate remodeling upholds integrity of all tissues in the body, giving rise to a steady background neo-epitope level in the systemic circulation. Chronic inflammation and fibrosis trigger a significant increase in tissue remodeling resulting in the release of pathology-related neo-epitopes above normal background levels. By assessing neo-epitopes as opposed to full length proteins, the signal-to-noise ratio may be improved by utilizing post-translational modifications, in this case proteolytic cleavage, that are associated with pathology [42, 43]. As with many systemic biomarkers, these neo-epitopes may originate from other organ systems than the lung, especially in relation to significant comorbidities that may affect tissue turnover. However, as COPD may be viewed as a systemic disease, the possible contribution from other affected organs may provide an assessment of the overall burden of disease.

It is intriguing that emphysema seems to be associated with lower levels of ECM remodeling while mortality is associated with higher levels. These results indicate that different pathological processes are related to distinct processing of the ECM leading to different levels of neo-epitopes. Acute exacerbations of COPD are generally associated with higher levels of remodeling [28], and individuals with lung cancer also show accelerated remodeling [44]. Emphysema seems to represent a different pathological process leading to a different pattern of remodeling. Clearly, more research is needed in this area to understand the specific mechanisms involved in pathological tissue turnover.

The recent qualification by the United States Food and Drug Administration of plasma fibrinogen as a drug development tool for enrichment of clinical trials in COPD, using exacerbations or all-cause mortality as endpoints [45–47], has helped pave the way for the use of validated biomarkers in clinical trials of COPD. Interestingly, in the present study several of the novel biomarkers, as well as CRP, performed better than fibrinogen for the prediction of all-cause mortality. The association of plasma fibrinogen with COPD has been seen in several studies [48, 49]. Although plasma fibrinogen is qualified as a biomarker for mortality in COPD, a recent review by Sin et al. concludes that none of the biomarkers investigated thus far have sufficient performance characteristics or specificity to be a clinically useful biomarker in COPD [50]. The ideal biomarker is stable over time and is related to the pathological process. As the COPD population is very heterogeneous, it seems likely that biomarkers reflecting different pathological processes may be needed for monitoring progression in different subtypes of COPD. Thus, the need of tools to assess disease activity is still a major limitation to the development of novel therapeutic approaches in COPD, as well as in the management and prognosis of disease [30, 50]. Biomarkers with these qualities would aid personalized health care and could have the potential to improve patient care tremendously [30].

Limitations to this study include the low number of deceased subjects, reducing statistical power. In spite of this, we found significant associations with mortality. The individuals included in this study are a subpopulation of the full ECLIPSE study comprising the study participants that progress the least and the most in terms of FEV_1_ decline during the study period. This is not the optimal population to study mortality in, and may explain the low number of events in this population. Furthermore, the present results need to be confirmed in an independent cohort to determine if the associations are applicable to the general COPD population.

## Conclusions

In conclusion, with this study we have demonstrated that mortality in individuals with COPD is associated with elevated levels of serological biomarkers of collagen remodeling. These biomarkers were prognostic for all-cause mortality, suggesting that a high remodeling rate is related to increased risk of mortality. The increased degradation of collagens found in the interstitial matrix of the lungs, indicate that ECM turnover is important in the pathology of COPD.

## Additional file

Additional file 1: Members of the ECLIPSE Steering and Scientific Committees and list of ECLIPSE Investigators. (DOCX 101 kb)

## Abbreviations

6MWD: 6-min walking distance
ADAMTS: A disintegrin and metalloproteinase with thrombospondin motifs
AUC: Area under the ROC curve
BGM: Degradation fragment of biglycan generated by MMPs
BM: Basement membrane
BMI: Body mass index
BODE: BMI obstruction, dyspnea, and exercise index
C1M: Degradation fragment of collagen type I generated by MMPs
C3A: Degradation fragment of collagen type III generated by ADAMTS
C3M: Degradation fragment of collagen type III generated by MMPs
C4M: Degradation fragment of collagen type IV generated by MMPs
C6M: Degradation fragment of collagen type VI generated by MMPs
CI: Confidence interval
COPD: Chronic obstructive pulmonary disease
CRMP: Degradation fragment of CRP generated by MMPs
CRP: C-reactive protein
ECLIPSE: Evaluation of COPD Longitudinally to Identify Predictive Surrogate End-points
ECM: Extracellular matrix
ELM7: Degradation fragment of elastin generated by MMP-7
EL-NE: Degradation fragment of elastin generated by neutrophil elastase
FEV_1_: Forced expiratory volume in 1 second
FVC: Forced vital capacity
IM: Interstitial matrix
MMP: Matrix metalloproteinase
Pro-C6: C5 domain of collagen type VI released during formation
ROC: Receiver operating characteristics
SD: Standard deviation
SEM: Standard error of mean
