# Supplementary material for: High levels of biomarkers of collagen remodeling are associated with increased mortality in COPD – results from the ECLIPSE study
Source: Respir Res. 2016 Oct 4;17:125. doi: 10.1186/s12931-016-0440-6 (PMC5050854; doi:10.1186/s12931-016-0440-6)
Supplement: Additional file 1: — Members of the ECLIPSE Steering and Scientific Committees and list of ECLIPSE Investigators. (DOCX 101 kb) [file 12931_2016_440_MOESM1_ESM.docx]

**Supplementary Appendix**

1. Members of the ECLIPSE Steering and Scientific Committees
2. List of ECLIPSE Investigators
3. Members of the ECLIPSE steering and scientific committees and the study investigators are as follows. **ECLIPSE Steering Committee:** H. Coxson, L. Edwards, R. Tal-Singer, D. Lomas, W. MacNee, E. Silverman, C. Crim, J. Vestbo, J. Yates. **ECLIPSE Scientific Committee:** A. Agusti, P. Calverley, B. Celli, C. Crim, B. Miller, W. MacNee, S. Rennard, R. Tal-Singer, E. Wouters, J. Yates.
4. **ECLIPSE Investigators:** Bulgaria: Y. Ivanov, Pleven; K. Kostov, Sofia. Canada: J. Bourbeau, Montreal; M. Fitzgerald, Vancouver, BC; P. Hernandez, Halifax, NS; K. Killian, Hamilton, ON; R. Levy, Vancouver, BC; F. Maltais, Montreal; D. O’Donnell, Kingston, ON. Czech Republic: J. Krepelka, Praha. Denmark: J. Vestbo, Hvidovre. The Netherlands: E. Wouters, Horn-Maastricht. New Zealand: D. Quinn, Wellington. Norway: P. Bakke, Bergen. Slovenia: M. Kosnik, Golnik. Spain: A. Agusti, J. Sauleda, P. de Mallorca. Ukraine: Y. Feschenko, V. Gavrisyuk, L. Yashina, Kiev; N. Monogarova, Donetsk. United Kingdom: P. Calverley, Liverpool; D. Lomas, Cambridge; W. MacNee, Edinburgh; D. Singh, Manchester; J. Wedzicha, London. United States: A. Anzueto, San Antonio, TX; S. Braman, Providence, RI; R. Casaburi, Torrance CA; B. Celli, Boston; G. Giessel, Richmond, VA; M. Gotfried, Phoenix, AZ; G. Greenwald, Rancho Mirage, CA; N. Hanania, Houston; D. Mahler, Lebanon, NH; B. Make, Denver; S. Rennard, Omaha, NE; C. Rochester, New Haven, CT; P. Scanlon, Rochester, MN; D. Schuller, Omaha, NE; F. Sciurba, Pittsburgh; A. Sharafkhaneh, Houston; T. Siler, St. Charles, MO; E. Silverman, Boston; A. Wanner, Miami; R. Wise, Baltimore; R. ZuWallack, Hartford, CT.
